# Integration of Gas Exchange With Metabolomics: High-Throughput Phenotyping Methods for Screening Biostimulant-Elicited Beneficial Responses to Short-Term Water Deficit

**DOI:** 10.3389/fpls.2021.678925

**Published:** 2021-06-01

**Authors:** Giulia Antonucci, Michele Croci, Begoña Miras-Moreno, Alessandra Fracasso, Stefano Amaducci

**Affiliations:** ^1^Department of Sustainable Crop Production, Università Cattolica del Sacro Cuore (UCSC), Piacenza, Italy; ^2^Department for Sustainable Food Process, Research Centre for Nutrigenomics and Proteomics, Università Cattolica del Sacro Cuore, Piacenza, Italy

**Keywords:** water stress, biostimulant, GAMM, metabolomics, tomato, gas exchange, glycinebetaine, climate change

## Abstract

Biostimulants are emerging as a feasible tool for counteracting reduction in climate change-related yield and quality under water scarcity. As they are gaining attention, the necessity for accurately assessing phenotypic variables in their evaluation is emerging as a critical issue. In light of this, high-throughput phenotyping techniques have been more widely adopted. The main bottleneck of these techniques is represented by data management, which needs to be tailored to the complex, often multifactorial, data. This calls for the adoption of non-linear regression models capable of capturing dynamic data and also the interaction and effects between multiple factors. In this framework, a commercial glycinebetaine- (GB-) based biostimulant (Vegetal B60, ED&F Man) was tested and distributed at a rate of 6 kg/ha. Exogenous application of GB, a widely accumulated and documented stress adaptor molecule in plants, has been demonstrated to enhance the plant abiotic stress tolerance, including drought. Trials were conducted on tomato plants during the flowering stage in a greenhouse. The experiment was designed as a factorial combination of irrigation (water-stressed and well-watered) and biostimulant treatment (treated and control) and adopted a mixed phenotyping-omics approach. The efficacy of a continuous whole-canopy multichamber system coupled with generalized additive mixed modeling (GAMM) was evaluated to discriminate between water-stressed plants under the biostimulant treatment. Photosynthetic performance was evaluated by using GAMM, and was then correlated to metabolic profile. The results confirmed a higher photosynthetic efficiency of the treated plants, which is correlated to biostimulant-mediated drought tolerance. Furthermore, metabolomic analyses demonstrated the priming effect of the biostimulant for stress tolerance and detoxification and stabilization of photosynthetic machinery. In support of this, the overaccumulation of carotenoids was particularly relevant, given their photoprotective role in preventing the overexcitation of photosystem II. Metabolic profile and photosynthetic performance findings suggest an increased effective use of water (EUW) through the overaccumulation of lipids and leaf thickening. The positive effect of GB on water stress resistance could be attributed to both the delayed onset of stress and the elicitation of stress priming through the induction of H_2_O_2_-mediated antioxidant mechanisms. Overall, the mixed approach supported by a GAMM analysis could prove a valuable contribution to high-throughput biostimulant testing.

## Introduction

Adaptation to climate change is becoming central to the conversation about water management for agriculture (Iglesias and Garrote, 2015). Among the suggestions to improve resiliency and adaptive capacity, the most cited approach is the introduction of drought-resistant crops. While this can be achieved through classical breeding and biotechnology, such efforts have so far produced little results (Nuccio et al., 2018). Moreover, in the second case, even when plants are successfully genetically modified (GM), resistant plants typically represent a restricted number of crops. In this sense, a technology applicable to multiple crops in multiple locations would represent a desirable alternative: as pointed out by Del Buono (2020), biostimulants could represent a sustainable measure to foster the resilience of cropping systems under limited water conditions. The earliest response of plants to drought is represented by stomatal closure, which results in the inhibition of photosynthesis (Michaletti et al., 2018), and therefore leads to CO_2_ uptake and concentration reduction (Medrano et al., 2002). Multiple osmolytes can be found among biostimulant constituents targeting water stress resistance in plants, such as glycinebetaine (GB). GB not only acts as an osmoregulator but also stabilizes the structures and activities of enzymes and protein complexes *via* detoxification of reactive oxygen species (Papageorgiou et al., 1991; Papageorgiou and Murata, 1995), while maintaining the integrity of membranes against the damaging effects of excessive salt (Tang et al., 2014, Tian et al., 2017, Yang and Lu, 2005; Mbarki et al., 2018), cold (Quan et al., 2004), heat (Yang et al., 2006; Allakhverdiev et al., 2007), freezing (Razavi et al., 2018; Wang et al., 2019), and also drought (Ma et al., 2006). The role of GB in plant resistance to abiotic stress has been widely investigated and documented (Gorham, 1995; Sakamoto and Murata, 2002; Ashraf and Foolad, 2007; Chen and Murata, 2010; Huang et al., 2020), ranging from exogenous application to genetic engineering (Fariduddin et al., 2013), to its biosynthesis and the underlying molecular mechanisms behind in planta accumulation under stress (Annunziata et al., 2019). While it has been argued that the osmolyte accumulation does not entail positive effects on yield under drought conditions (Serraj and Sinclair, 2002), many reports demonstrate the positive effect of osmolyte accumulation generally (notably Blum, 2016) and GB specifically on plant growth and final yield. Among drought resistance mechanisms activated by GB, osmotic adjustment has been observed to enhance soil moisture extraction and, therefore, transpiration (Blum, 2016). At the same time, higher yielding plants are characterized by high stomatal conductance over time and higher transpiration (Blum, 2009). Indeed, Mäkelä et al. (1999) found that GB could enhance photosynthetic efficiency by reducing photorespiration and enhancing stomatal conductance in tomato plants grown under drought and salinity. This resulted in increased net photosynthesis of stressed plants. This is of particular relevance since tomato plants (*Solanum lycopersicum* L.) do not naturally accumulate GB (Wyn Jones and Storey, 1981). GB foliar application was also found to increase the yield of tomato plants under saline or heat stress (Mäkelä et al., 1998). Likewise, Agboma et al. (1997a,b) found that exogenous application of GB was indeed involved in the maintenance of a higher yield in maize and sorghum grains and tobacco leaves and resulted in improved water-use efficiency. GB was found to have a positive effect on yield also on soybean (Agboma et al., 1997b), common beans (Xing and Rajashekar, 1999), wheat (Agboma et al., 1997a), sunflower (Hussain et al., 2008), and cotton (Ahmad et al., 2014). Moreover, Park et al. found that genetically engineered GB (2004) and exogenously applied GB (2006) increased the tolerance of tomato plants to chilling stress. Interestingly, they suggest that, in addition to its macromolecule and membrane protecting action, GB-enhanced chilling tolerance might imply stress priming through the induction of H_2_O_2_-mediated antioxidant mechanisms.

According to Fleming et al. (2019), scientific recognition of the potential of biostimulants has not grown as fast as the interest from industry. This has been due to limited fundamental research of their modes of action and the speed at which new multi-compound products have entered the market. In the investigation of biostimulant activity, the necessity of accurate assessment of phenotypic variables is emerging as a critical issue (Rouphael et al., 2018) while at the same time the combined phenotypic-omic approach is gaining momentum. In recent years, emerging digital technologies such as sensors, automatic image acquisition, and the connected algorithms and models have seen an increasing adoption, resulting in increasing volumes and complexity of data. Large-scale acquisition of data has allowed data interpretation to shift from a model-based to a data-based paradigm, by improving the possibility to generalize the data acquired and consequently allowing for an increase in model accuracy. On the flip side of the coin, the main challenge lies in data management: the huge amount of data generated needs to be handled both at the acquisition and analysis stage through proper, often custom, tools (Coppens et al., 2017). In this context, non-destructive phenotyping techniques characterized by high accuracy (high-throughput techniques) have gained popularity in the scientific community and have been successfully employed in plant breeding (Araus and Cairns, 2014; Halperin et al., 2017; Tardieu et al., 2017; Campbell et al., 2018; Mir et al., 2019), precision agriculture (Chawade et al., 2019), and biostimulant activity testing (Petrozza et al., 2014; Rouphael et al., 2018; Paul et al., 2019a,b). High-throughput phenotyping technologies have attracted attention for their potential in: (1) screening and monitoring multiple morpho-physiological traits; (2) time-series measurements, crucial in the acquisition and interpretation of high spatial and temporal resolution data; and (3) labor automation, time, and cost efficiency (Rouphael et al., 2018). Halperin et al. (2017) have proposed a platform, which uses a custom algorithm to select genotypes based on their abiotic stress resistance characteristics. The system coupled with single-leaf gas exchange acquisition provides high-resolution whole plant transpiration, biomass gain, stomatal conductance, and root flux. As pointed out by Teklić et al. (2020), in addition to the investigation of the effect of biostimulants on plant stress response, there is a growing necessity to elucidate stress and interactions of biostimulants in terms of metabolic changes. To further explore the variation in physiological traits, the integration of phenotyping data and omics data represents the next frontier and a promising tool to bridge the phenotype–genotype gap (Coppens et al., 2017) and to understand dynamic plant stress responses in a changing environment (Gosa et al., 2019). Specifically, high-throughput phenotyping data in combination with metabolomics have already been successfully applied to biostimulant testing (Lucini et al., 2015; Paul et al., 2019a,b; Rouphael et al., 2020). In metabolomics, two commonly used analytical strategies can be distinguished: untargeted and targeted strategies. Untargeted metabolomics, by aiming to detect as many metabolites as possible in a biological sample, is particularly suited to identifying metabolite abundance variations connected to either environmental stimuli or genetic variations (Cheng et al., 2018). Metabolomics has a recognized potential to provide significant insights into the mechanisms of the stress response (Shulaev et al., 2008; Arbona et al., 2013) by identifying different compounds, such as the molecules involved in stress acclimation (e.g., secondary metabolites) and by-products of stress metabolism, and has been successfully applied to the investigation of biostimulant action in general (Lucini et al., 2015, 2018; Paul et al., 2019a,b) and especially under abiotic stresses (Nephali et al., 2020). Lastly, responding to the necessity to analyze the volumes and complexity of data produced through high-throughput phenotyping, non-linear regression models are emerging. These models need to be capable of capturing dynamic data, often time series, as well as the interaction and effects between multiple factors (Ohana-Levi et al., 2020). Among these, generalized additive mixed models (GAMMs) have been successfully featured in several applied science fields (Murase et al., 2009; Zuur et al., 2009; Pedersen et al., 2019; Boswijk et al., 2020; Ohana-Levi et al., 2020) including ecology at large and plant ecophysiology. In particular, Ohana-Levi et al. (2020) highlighted the potential of GAMs to model non-linear relationships between evapotranspiration (ETc) drivers and evaluating their impacts on grapevine transpiration.

In light of this, GB is particularly fit to test the suitability and accuracy of GAMMs in modeling dynamic plant response to drought: widely investigated, its protective action on the photosystem is documented for several abiotic stresses (Huang et al., 2020). At the same time, while available research concentrates mostly on the effect of GB application on yield traits and cell-level effects (Annunziata et al., 2019), a correlation between GB treatment and modifications in photosynthetic rates has received little attention: published research mainly concerns enhanced photosynthetic performance under salt stress (Yang and Lu, 2015; Hamani et al., 2020) and drought (Nawaz and Wang, 2020). Likewise, the effects of GB on water-use efficiency (WUE) have been scarcely investigated (Ahmed et al., 2019) and although both of its abundance (natural or GM) in plant tissues and its exogenous application have been widely linked to stress response, the effects of exogenous application of GB on the metabolomic profile of leaves are yet to be investigated.

Based on the current literature, the hypothesis tested here was whether a biostimulant (GB-based) treatment can be efficiently modeled through GAMM and whether the treatment would result in tangibly different metabolite expression profiles, thereby completing the information derived from GAMM. In order to achieve this, the duration and dynamics of the effect on photosynthesis, transpiration, and WUE were investigated, jointly with a snapshot analysis of metabolic profile.

## Materials and Methods

### Plant Material and Growing Conditions

The experiments were conducted in a greenhouse and the metabolomics analysis in the laboratory of the Department of Sustainable Crop Production of the Università Cattolica del Sacro Cuore, Piacenza. Tomato seeds (H1281 variety, Heinz) were directly sown in a greenhouse at 20°C at 2 cm in a seeding tray (35-ml cell plug) containing a commercial complete mixture of sand—blonde peat—humus. Seeds were kept in dark conditions for 5 days until germination, thereafter they were provided with a PPFD of 800 μmol m^−2^ s^−1^ through light-emitting diode (LED) lighting. Seven days after emergence (DAE), 50 uniformly developed seedlings, at second true leaf unfolded (BBCH 12), were transplanted in 2.3-L pots filled with a commercial complete mixture of sand—blonde peat—humus (1,000 ± 1 g). Plants were fertilized at a transplant with 40 ml of a complete commercial solution (COMPO, Concime Liquido Universale, Bologna, Italy) at 1.05% w/v (7% N; 5% P; 6% K; 0.01% B; 0.004% Cu; 0.04% Fe; 0.02% Mn; 0.001% Mo; and 0.002% Zn). Fertilization was provided every 2 weeks. The pots were placed under LED lamps to provide a PPFD of 800 μmol m^−2^ s^−1^ to the top canopy with a photoperiod of 16 h light and 8 h dark. Pots were watered to field capacity (FC) every second day. The temperature was not controlled and in the range between 35°C during the day and 8°C during the night (19.5°C average). Of the 50 plants, 32 homogeneously developed plants were selected, of which 12 plants were destined for gas exchange analysis and 20 for metabolomics analysis, while the rest was discarded.

The experiment was designed as a factorial combination of irrigation (water-stressed and well-watered) and biostimulant treatment (treated and control). The water-stressed plants were allowed to dry down for three consecutive days by the withdrawal of irrigation. Thereafter, all plants were irrigated. Gas exchange analysis was carried out on three replicates for each treatment, for a total of 12 plants while metabolomics analyses were carried out on five replicates per each treatment, totaling 20 plants. Plants dedicated to gas exchange analyses were kept in the gas exchange acquisition system for 9 days until the end of the experiment (3 initial days to adapt to the ventilation and lighting, followed by dry down) while plants dedicated to metabolomics analysis were kept in the greenhouse under a tunnel replicating the conditions of the gas exchange acquisition system (same air inlet and LED lighting).

### Biostimulant Characteristics and Treatment

The day before the beginning of the gas exchange analysis, the pots were irrigated to saturation and allowed to drain overnight. The treatment with the biostimulant was carried out on 36 DAE on one half of the plant while the other half was sprayed with distilled water. Biostimulant was sprayed at a rate of 6 kg/ha, with a dilution of 300 L/ha to a total of 10 g/plant. Dose and dilution were chosen based on commercial use of the product (Vegetal B60®, ED&F MAN Liquid Products Italia, Bologna, Italy). Vegetal B60® is an organic product extracted exclusively from sugar beet without any added chemical additives. It contains 30% of GB and 5% of L–amino acids, 5% of total organic nitrogen, and 12% of organic carbon.

All pots were sealed in plastic bags fitted around the base of each plant stem to prevent soil evaporation. Well-watered plants were kept at 80% FC throughout the experiment, while for water -stressed plants irrigation was interrupted 3 days after the treatment (on 39 DAE). About 200 g of water were reintegrated to WS plants on 41 DAE, 2 days after irrigation was stopped, to contrast the high rate of soil drying and allow for a longer dry-down period and a more gradual onset of drought stress (a transpiration exceeding 200 g d^−1^ would have brought the plant at wilting point on DAE 41). Leaf area (LA) of each plant was calculated every 3 days from the start of stress imposition (39 DAE). Leaves were placed on squared paper (square of side 0.5 cm, used as the reference of known size) reinforced with a rigid base and photographed with a phone camera, taking care that no leaves overlapped. Images were then processed by using ImageJ (Schneider et al., 2012) to extract LA and compute the total LA of each plant.

### Gas Exchange Analysis

Gas exchange was evaluated through a semiautomated multichamber whole-canopy system [slightly modified from the system previously described in Fracasso et al. (2017)]. In particular, the system computes net photosynthetic rate (*Pn*), transpiration rate (E), and WUE. Every pot was measured every 12 min for a total of 120 measurements per day. *Pn* (μmol s^−1^ m^−2^) and E (mmol s^−1^ m^−2^) were calculated based on flow rates and CO_2_ and water vapor differentials by using the formula provided in Long and Hallgren (1985). Data were acquired 24 h per day at intervals of 1 min. The semiautomated multichamber system is composed of 12 adjustable open chambers connected to a CIRAS-DC double channel absolute CO_2_/H_2_O IR gas analyser (PP-System, Amesbury, MA, USA). Air drawn at 3 m above ground from outside the greenhouse is collected in a buffer tank (0.44 m^3^ capacity) to ensure the stability of inlet CO_2_ concentration and then, forced by one centrifugal blower (Vorticent C25/2M Vortice, Milan, Italy), to distribute air to the chambers through 50-mm-diameter rigid plastic pipes. The air flow rate of each chamber is controlled by a baffle and is constantly measured at least 50 cm downstream of the baffle itself with digital flowmeters (50 Pa D6F-PH0026AD1, OMRON, Kyoto, Japan) according to a flow-restriction method (Osborne, 1977). Each chamber is connected to a set of 12 solenoid valves in series (model 177 B04/Z610, Sirai, Bussero, Italy) through a sampling tube (Ø 10 mm). Air sampling is switched from one chamber to another at programmed time intervals (Raspberry Pi B+, Raspberry Pi Foundation, Northants, UK). In order to ensure air flushing inside the sampling tubes and the complete air exchange inside CIRAS-DC, rotary vane pumps (model G 6/01-K-LCL; Gardner, Denver Thomas, Pucheim, Germany) with 33.3 cm^3^ s^−1^ of flow rate were added before CIRAS-DC. Both the inlet air temperature and the air temperature of each chamber (outlet) were measured by using digital temperature sensors (Dallas DS18B20, Dallas Semiconductor Corp., Dallas, TX, USA).

On 36 DAE, 12 plants were transferred under the gas exchange acquisition system and were kept in the system for 9 days until the end of the experiment (as mentioned above) while 20 plants were kept under a tunnel replicating the conditions of the gas exchange acquisition system and were sampled for metabolomics analyses. Both the tunnel and the gas acquisition system were provided with the same air inlet, namely forced air collected from outside (as described above), and the same LED lighting, namely 800 μmol m^−2^ s^−1^ PPFD.

### Sample Harvest and Metabolomic Analysis

Gas exchange was measured throughout the duration of the stress: as soon as the *Pn* of stressed plants started decreasing, plants were sampled for metabolomics. Sampling of the 20 plants kept under the tunnel replicating the conditions of the gas acquisition system was carried out on 42 DAE. The second and third fully expanded leaves from the top of each plant were excised and dipped into liquid nitrogen. Samples were kept at −20°C and subsequently analyzed. Plant samples were homogenized in pestle and mortar by using liquid nitrogen and extracted as previously reported (Paul et al., 2019b). Briefly, an aliquot (1.0 g) was extracted in 10 ml of 0.1% HCOOH in 80% aqueous methanol by using an Ultra-Turrax (Ika T-25, Staufen, Germany). The extracts were centrifuged (12,000 g) and filtered into amber vials through a 0.22-μm cellulose membrane for analysis. Thereafter, metabolomic analysis was carried out by ultra-high performance liquid chromatograph (UHPLC) coupled to a quadrupole-time-of-flight mass spectrometer (UHPLC/QTOF-MS). The metabolomic facility included a 1,290 ultra-high-performance liquid chromatograph, a G6550 iFunnel Q-TOF mass spectrometer, and a JetStream Dual Electrospray ionization source (all from Agilent Technologies, Santa Clara, CA, USA). The untargeted analysis was carried out as previously described (Rouphael et al., 2016). Briefly, reverse-phase chromatography was carried out on an Agilent PFP column (2.0 × 100 mm, 3 μm) and by using a 33-min linear elution gradient (6–94% acetonitrile water, with a flow of 200 μl min^−1^ at 35°C). The mass spectrometric acquisition was done in SCAN (100–1,000 m/z) and positive polarity (Pretali et al., 2016). Quality controls (QCs) were analyzed in data-dependent MS/MS mode by using 12 precursors per cycle (1 Hz, 50–1,200 m/z, positive polarity, active exclusion after 2 spectra), with collision energies of 10, 20, and 40 eV for collision-induced decomposition.

Raw spectral data were processed by using Agilent Profinder B.07 software, (Santa Clara, CA, USA) as described in Miras-Moreno et al. (2020). The PlantCyc 12.6 database (Plant Metabolic Network; Release: April 2018) was used to putatively annotate compounds according to Level 2 with reference to the COSMOS Metabolomics Standards Initiative (Salek et al., 2015). QCs were analyzed by using the MS-DIAL 3.98 (RIKEN Center for Sustainable Resource Science: Metabolome Informatics Research, Yokohama, Japan) to compare the MS/MS spectra to the publicly available MS/MS experimental spectra built in the software (e.g., MoNA) (Tsugawa et al., 2015).

### Data Analysis

#### Gas Exchange Curve Fitting Using GAM(M)

The experiment was evaluated from the day prior to stress imposition (38 DAE) for three consecutive days of water stress (DAE 39-41) until the start of stress recovery (42 DAE). Statistical analyses on gas exchange data were carried out *via* GAMMs. GAMMs are regression models, which allow for modeling non-linear regressions (Wieling, 2018). Unlike linear models (1), which feature a sum of linear terms, GAMMs (2) are characterized by a sum of smooth functions. GAMMs are used to estimate smooth functional relationships between predictor variables and the response. GAMM data fitting is characterized by a penalized fit: the fit of the data is balanced with the complexity of the model by penalizing wiggliness, namely the deviation from total smoothness, and thus avoiding overfitting (Pedersen et al., 2019).

(1) $$Y_{ij=}\beta_{0}+\sum \beta_{j}x_{i}+\in_{ij}$$

(2) $$Y_{ij=}\beta_{0}+\sum f_{j}(x_{i})+\in_{ij}$$

Among the multiple advantages of this approach is the possibility to handle the complexity of the data without oversimplifying them, the determination of the relationship between the dependent variable and the predictors as a function of the algorithm, which can be linear but is not necessarily linear, the inclusion of multiple numeric predictors and the possibility to include autoregressive AR(1) error model for Gaussian models in order to handle the autocorrelation component of the error (van Rij et al., 2019). The inclusion of autocorrelation is particularly relevant in time-series data sets, where each datapoint is clearly correlated to (and therefore dependent on) the previous and the next datapoints. Therefore, this analysis is particularly useful to data sets characterized by dynamic and time-series data (Boswijk et al., 2020).

After Wieling (2018), the analysis started from a simple generalized additive model, the sophistication of which was progressively increased and extended step-by-step. While one would normally directly choose the model reflecting the hypothesis, this approach was chosen to shed light on the process using generalized additive modeling.

Net photosynthesis (*Pn*), transpiration rate (E), and WUE were used as the response variable for the following series of GAM(M)s:

a. Sole irrigation as a fixed factor, including a smooth for day-of-treatment and hour in the day, based on treatment.
b. Irrigation treatment and biostimulant treatment as fixed factors, no interaction between the factors. Including a smooth for day-of-treatment and hour in the day, based on both treatments.
c. Irrigation treatment and biostimulant treatment as fixed factors, interaction between the factors. Including a smooth for day-of-treatment and hour in the day, based on both treatments.
d. Introduction of tensor product-based smooths between hour and day.
e. Inclusion of a smooth for day-of-treatment and hour in the day, based on both treatments, and allowing for random effect per individual.
f. Addition of non-linear random effects per individual for day-of-treatment.
g. Introduction of individual-based autocorrelation.

Models d:g are nested within model c, allowing for comparison of methods using log-likelihood/F tests. This allowed us to evaluate Akaike information criterion (AIC) changes among models and, thus, to find the model with the most explanatory power given the degrees of freedom, while at the same time assessing whether better or worse models explained significantly different amounts of the deviance in the data. Statistical tests were performed by using the software R version 4.0.2 (R Core Team, 2020). GAMM models were fitted in R by using a cubic regression spline smoother, with the package itsadug (van Rij et al., 2020). Mixed model selection, fitting, and validation followed (Zuur et al., 2009). Biostimulant and stress treatment factors were used in mixed linear models for hypothesis testing.

#### Chemometric Interpretation of Metabolites

Chemometric interpretation of metabolites was performed by using Mass Profiler Professional B.12.06 from Agilent (Santa Clara, CA, USA) as previously described (Corrado et al., 2020). The unsupervised hierarchical cluster analysis (HCA—distance measure: Euclidean; clustering algorithm: Ward's) was produced based on the normalized molecular features. The supervised orthogonal partial least squares-discriminant analysis (OPLS-DA) was carried out with SIMCA 16 (Umetrics, Umeå, Sweden) at default parameters. CV-ANOVA (*p* < 0.01) and permutation testing (*n* = 100) were also applied to validate and to exclude overfitting. Goodness-of-fit R2Y and goodness-of-prediction Q2Y were also calculated from the OPLS-DA model. Outliers were investigated according to Hotelling's T2 (95 and 99% confidence limit for the suspect and strong outliers, respectively). The most discriminant compounds in separation were selected by performing the variables importance in projection (VIP) analysis.

Thereafter, differential compounds were investigated through Volcano plot analysis, combining fold-change (FC > 2) and ANOVA (*p* < 0.0, 1, Bonferroni multiple testing correction) and were then uploaded into the Omic Viewer Pathway Tool of PlantCyc (Stanford, CA, USA) to identify pathways affected by the treatments as in Caspi et al. (2013).

## Results

### Gas Exchange Analysis

On 36 DAE, 12 plants were transferred under the gas exchange acquisition system and were kept in the system for 9 days until the end of the experiment. Plants were treated with a factorial combination of irrigation (well-watered and water-stressed) and biostimulant treatment (treated and control). Water-stressed plants were allowed to dry down for three consecutive days by the withdrawal of irrigation. Gas exchange data were processed *via* GAMMs. GAMMs are non-linear regression models which are characterized by a sum of smooth functions. Data were analyzed by building models of growing complexity (Table 1, model a–g), as previously explained. The regression models allowed to explore the dynamics of the influence of the biostimulant and water treatments on photosynthetic performance.

**Table 1:** Characteristics of the nine compared models of influence on photosynthesis.

**Model**	**Resid. Df**	**Resid. Dev**	**AIC**	**dAIC**	**Dev. Expl (%)**
a	3391.454	11624.1257	13974.67	5006.285	61.5
b	3378.586	10206.6696	13552.31	4583.922	66.2
c	3377.564	9648.388	13361.94	4393.553	68
d	3265.717	7618.79372	12724.36	3755.977	74.7
e	3231.964	4454.29886	10950.84	1982.455	85.2
f	3182.686	3307.85823	10024.21	1055.83	89
g	3214.175	3679.9617	8968.384	0	87.8

*Model names as in the text: a is the sole irrigation as a fixed factor, including a smooth for day-of-treatment and hour in the day, based on treatment. b has both irrigation and biostimulant treatment as fixed factors. c adds the interaction between the factors and includes a smooth for day-of-treatment and hour in the day, based on both treatments. d introduces tensor product-based smooths between hour and day. e includes a smooth for day-of-treatment and hour in the day, based on both treatments, and allowing for random effect per individual. f adds non-linear random effects per individual for day-of-treatment. g introduces individual-based autocorrelation. Resid.Df, residual degrees of freedom; Resid.Dev, residual deviance; dAIC, difference in AIC based on model g. Dev. Expl. (%) = deviance explained*.

Among the candidate GAMMs, the optimal model was model g, including Photosynthesis ~ irrigation treatment ^*^ biostimulant treatment and non-linear interactions between both treatments and the duration of the experiment (expressed in days) and the time of the day (expressed in hours), smooths for the duration of the experiment and the time of the day on both treatments and non-linear variability of the individuals over the duration of the experiment. Moreover, model g included autocorrelation for time over the individual. The model explained 87.8% of the total deviance with an Adj.*R*^2^ of 0.872. While the next best model, which did not include autocorrelation (model f), explained 89% of the deviance (Adj.*R*^2^ 0.883), when the models were confronted *via* AIC (Akaike, 1974), and model g scored consistently lower in terms of AIC score although bearing more degrees of freedom (Table 1).

The inclusion of the sole irrigation treatment factor (model a) explained 61.5% of the deviance in the data. The addition of the biostimulant treatment factor (model b) increased the deviance explained by 4.7%. The addition of further information increased the deviance explained as follows: the inclusion of the interaction between the fixed factors (model c) resulted in an additional 1.8%. The further introduction of tensor product-based smooths between hour and day (model d) added 6.7% deviance explained, while allowing for random effect per individual (model e) further added 10.5% to it. Lastly, the addition of non-linear random effects per individual for day-of-treatment (model f) resulted in 1.8% more deviance explained.

**Figure 1:**
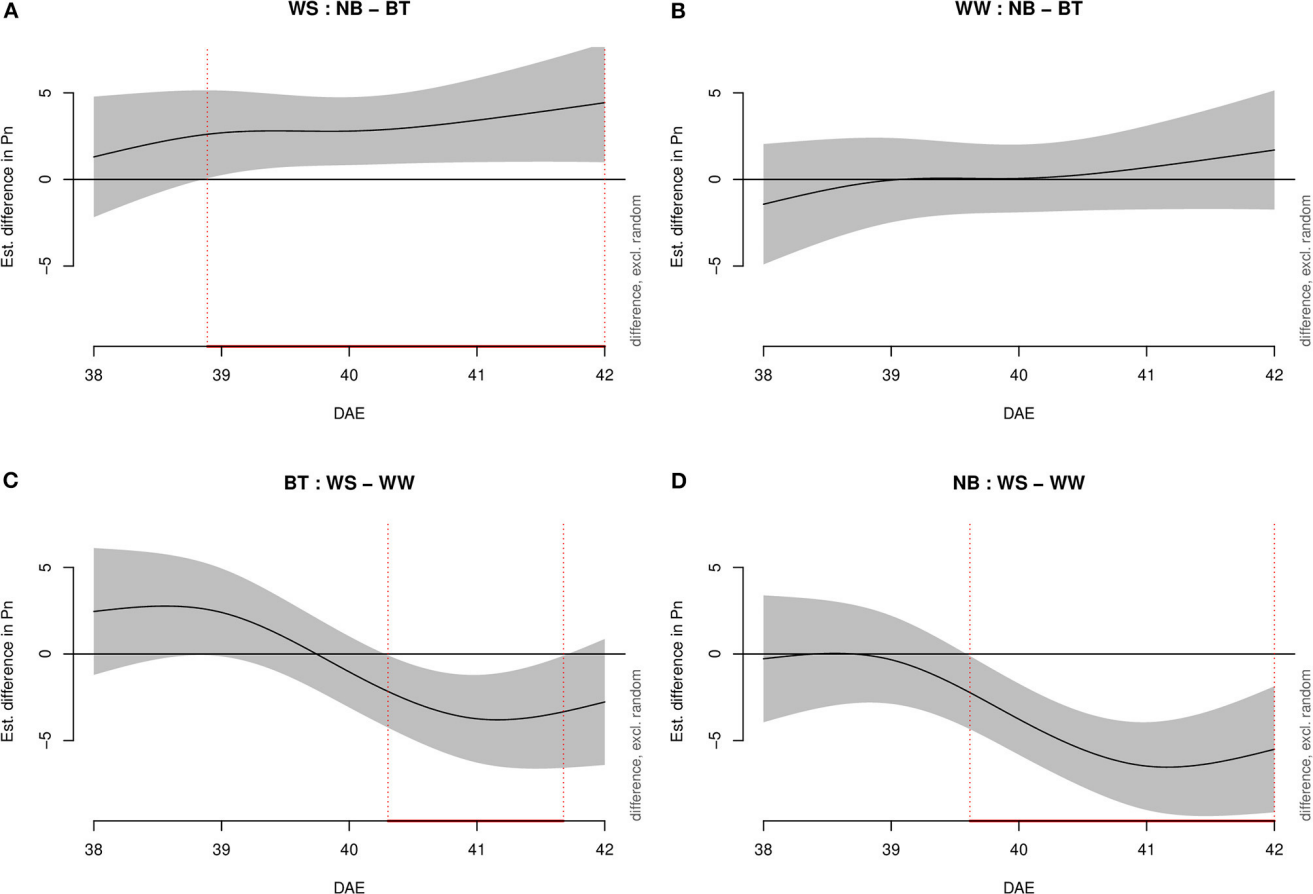
Graphs showing differences in *Pn* according to model g among all combinations of treatments (irrigation and biostimulant). Random effects were excluded. The baseline is represented by untreated plants above and by watered (control) plants below. The pointwise 95% CI is shown by a shaded band. When the shaded confidence band does not overlap with the *x*-axis (i.e., the value is significantly different from zero), this is indicated by a red line on the *x*-axis (and vertical dotted lines). **(A)** Water-stressed (WS), comparison between no biostimulant (NB) and biostimulant treatment (BT). **(B)** Well-watered (WW), comparison between NB and BT. **(C)** BT, comparison between WS and WW. **(D)** NB, comparison between WS and WW.

Under water-stressed conditions, model g highlighted statistically higher *Pn* (Figure 1) in plants treated with biostimulant compared to control plants starting shortly before 39 DAE and lasting throughout the experiment until 42 DAE. Conversely, under well-watered conditions, there was no significant difference between treatments. When considering biostimulant-treated (BT) plants, the difference between water-stressed and well-watered plants was significantly lower for water-stressed plants starting half 40 DAE to half 41 DAE. Lastly, well-watered and water-stressed untreated plants displayed a significant negative difference from mid-39 DAE to mid-42 DAE.

The influence of day-of-treatment (38–42 DAE), starting from the day prior to stress imposition, and hour was evaluated separately for each treatment (biostimulant and water treatment). Through the use of GAMMs, the significance of the effect of day and hour on biostimulant and water treatment was further investigated singularly for each dimension of both factors (biostimulant treated/control treated and water-stressed/well-watered) (Table 2). The results show that all the partial correlations were statistically significant for at least one of the two levels of the factors at the value of *p* < 0.001 level, except for the effect of hour and day of week on biostimulant treatment, which was significant at the value of *p* < 0.05. While the significance of the smooth terms does not provide information on whether the patterns are statistically significant, this suggests that each individual variable has a statistically significant influence on modeling the wiggliness of *Pn*, which in turn confirms the distance of *Pn* from a linear function.

**Table 2:** Influence of partial effects on *Pn* using generalized additive mixed modeling (GAMM) multivariate analysis.

**Partial effect**	**Edf**	***F***	***p*-value**
te(day_of_treatment, hour):WellWatered	1.819e+01	0.481	0.000509^***^
te(day_of_treatment, hour):WaterStressed	3.084e+01	5.554	<2e−16^***^
te(day_of_treatment, hour):NoBiostimulant	6.220e+00	0.219	0.000288^***^
te(day_of_treatment, hour):BiostimulantTreated	2.847e+01	1.636	<2e−16^***^
s(day_of_treatment): WellWatered	1.000e+00	2.872	0.090207
s(day_of_treatment): WaterStressed	3.803e+00	17.018	8.28e−12^***^
s(day_of_treatment): NoBiostimulant	9.612e−05	0.666	0.993127
s(day_of_treatment): BiostimulantTreated	3.415e+00	3.049	0.010673^*^
s(hour): WellWatered	1.632e+01	11.215	<2e−16^***^
s(hour): WaterStressed	6.051e+00	3.626	0.000573^***^
s(hour): NoBiostimulant	1.000e+00	1.258	0.262130
s(hour): BiostimulantTreated	5.880e+00	2.523	0.012176^*^
s(day_of_treatment, ID_plant)	3.805e+01	26.618	<2e−16^***^

* *significant difference at P < 0.05*,

*** *significant difference at P < 0.001*.

**Figure 2:**
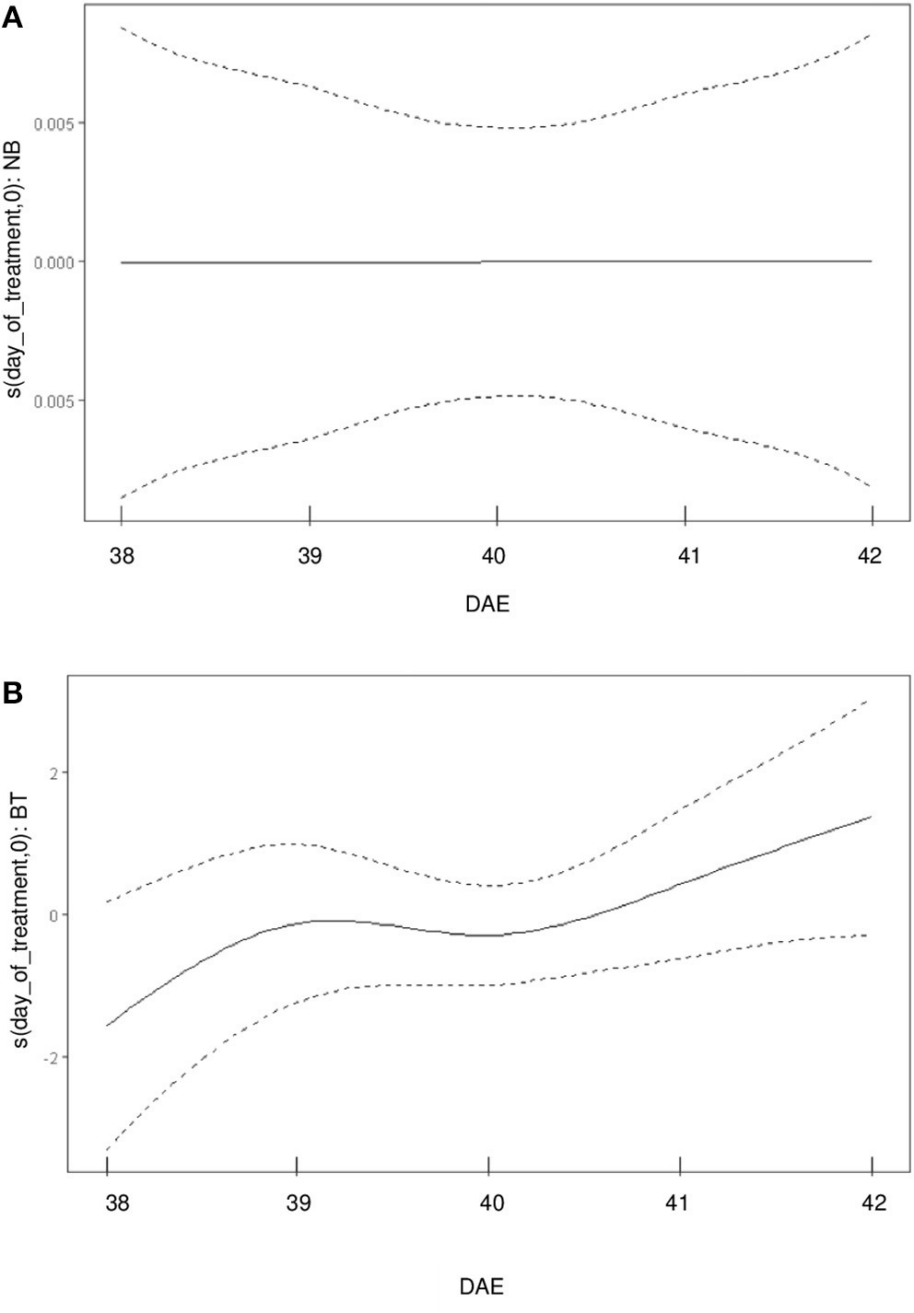
Uncoupling of the partial effects of BT/control treatment on the wiggliness of *Pn* curve. **(A)** partial effects of control treatment (NB) over the day of treatment [in days after emergence (DAE)] on *Pn*. **(B)** partial effects of BT over the day-of-treatment (in DAE) on *Pn*.

The partial effect (referred solely to biostimulant treatment) of biostimulant treatment on *Pn* observed for each day-of-treatment is of particular interest (Figure 2): while no effect can be detected for the control treatment (which is represented by a linear function), the biostimulant treatment has a detectable effect on the wiggliness of the *Pn* curve.

**Figure 3:**
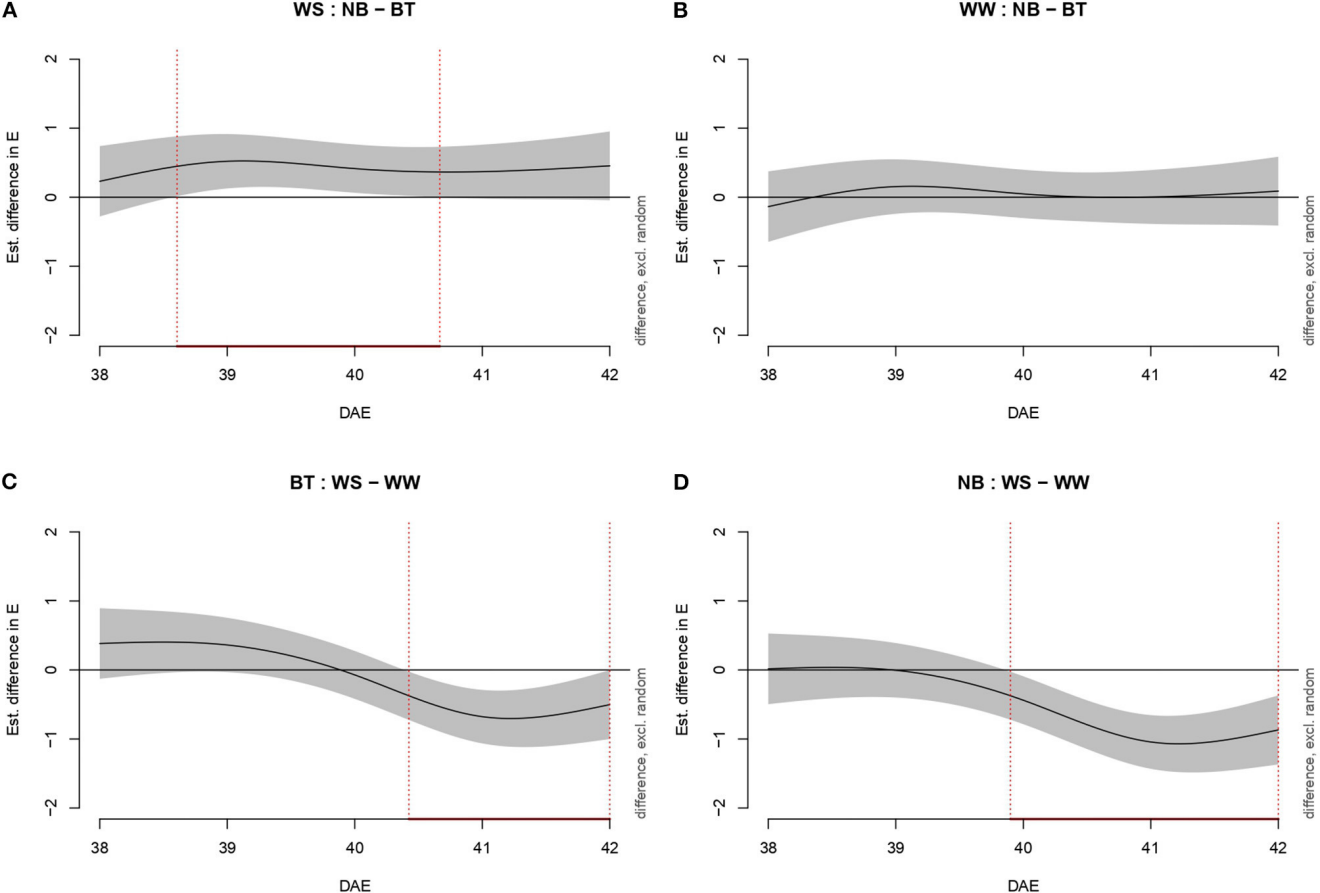
Graphs showing differences in transpiration (mmol m^−2^ s^−1^) according to model g among all combinations of treatments (irrigation and biostimulant). Random effects are excluded. The baseline above is represented by untreated plants, below by watered (control) plants. The pointwise 95% CI is shown by a shaded band. When the shaded confidence band does not overlap with the *x*-axis (i.e., the value is significantly different from zero), this is indicated by a red line on the *x*-axis (and vertical dotted lines). **(A)** WS, comparison between NB and BT. **(B)** WW, comparison between NB and BT. **(C)** BT, comparison between WS and WW. **(D)** NB, comparison between WS and WW.

Transpiration (E, mmol m^−2^ s^−1^) was evaluated through increasingly complex models, as *Pn*. Model g was once more the best fitting model, with 88.2% of deviance explained and 0.875 Adj.*R*^2^. As for *Pn*, water-stressed BT plants performed significantly better than untreated plants (Figure 3). In particular, the performance of BT plants was significantly higher from mid-38 DAE to mid-40 DAE. Conversely, non-stressed plants (biostimulant treated/untreated) did not show any significant difference in transpiration. Water-stressed BT plants had a higher E than well-watered plants from mid-41 DAE until mid-42 DAE. Untreated plants differed from late 39 DAE throughout the end of the experiment.

**Figure 4:**
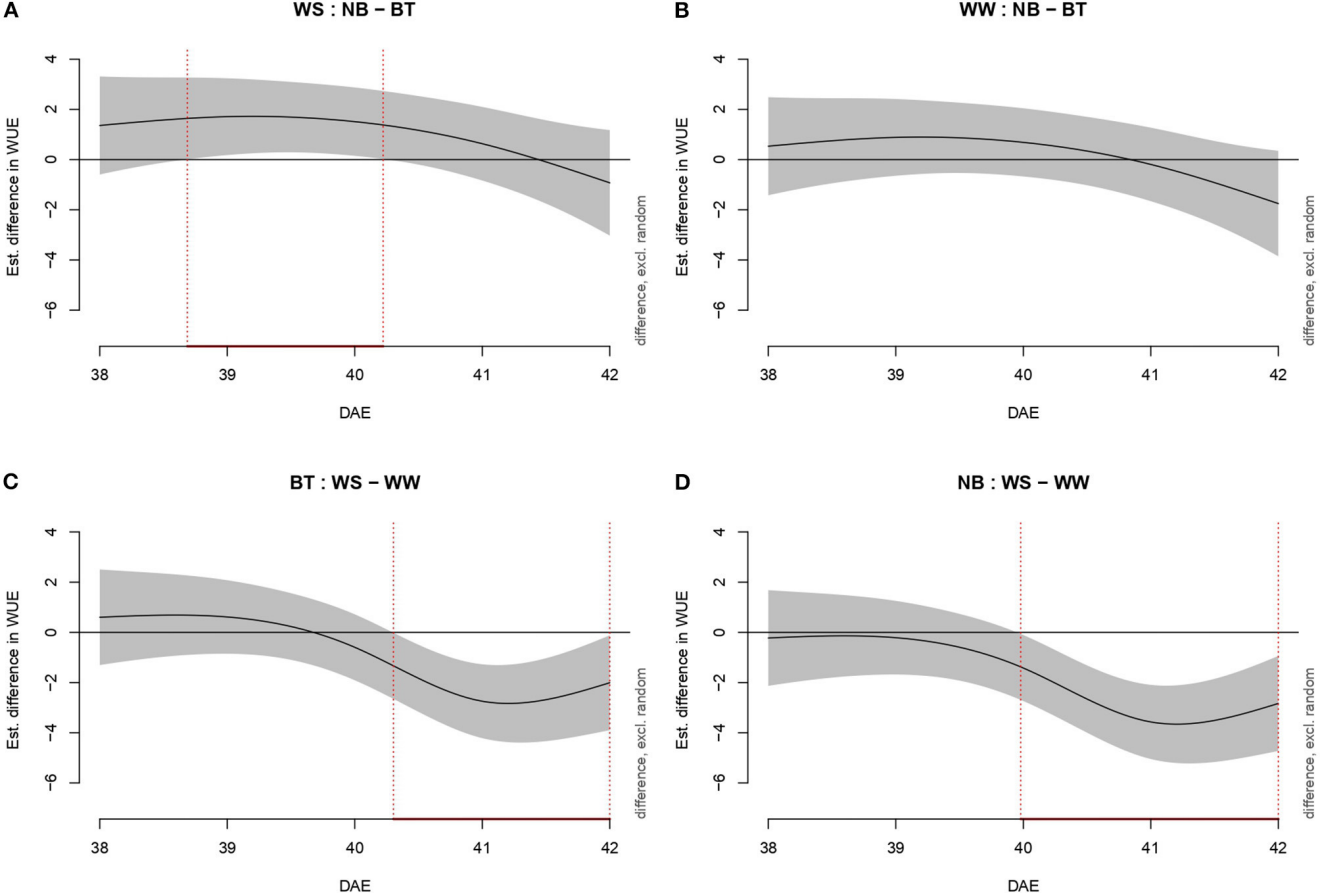
Graphs showing differences in water-use efficiency (WUE) among all combination of treatments (irrigation and biostimulant). Random effects are excluded. The baseline above is represented by untreated plants, below by watered (control) plants. The pointwise 95% CI is shown by a shaded band. When the shaded confidence band does not overlap with the *x*-axis (i.e., the value is significantly different from zero), this is indicated by a red line on the *x*-axis (and vertical dotted lines). **(A)** WS, comparison between NB and BT. **(B)** WW, comparison between NB and BT. **(C)** BT, comparison between WS and WW. **(D)** NB, comparison between WS and WW.

As for *Pn* and E, WUE was evaluated through increasingly complex models (Figure 4). Model g was confirmed as the best model, with 85.3% deviance explained and 0.843 Adj.*R*^2^.: As for *Pn* and E, no significant difference was highlighted among well-watered plants (both biostimulant treated and not). The difference in WUE between treated and untreated plants spanned from shortly before the day of stress imposition (39 DAE) to the third day (41 DAE). The duration of the difference among BT plants (well-watered/water-stressed) extended from early 40 DAE to 42 DAE while the difference among untreated well-watered and water-stressed plants went from 40 DAE to 42 DAE.

### Untargeted Metabolomics

**Figure 5:**
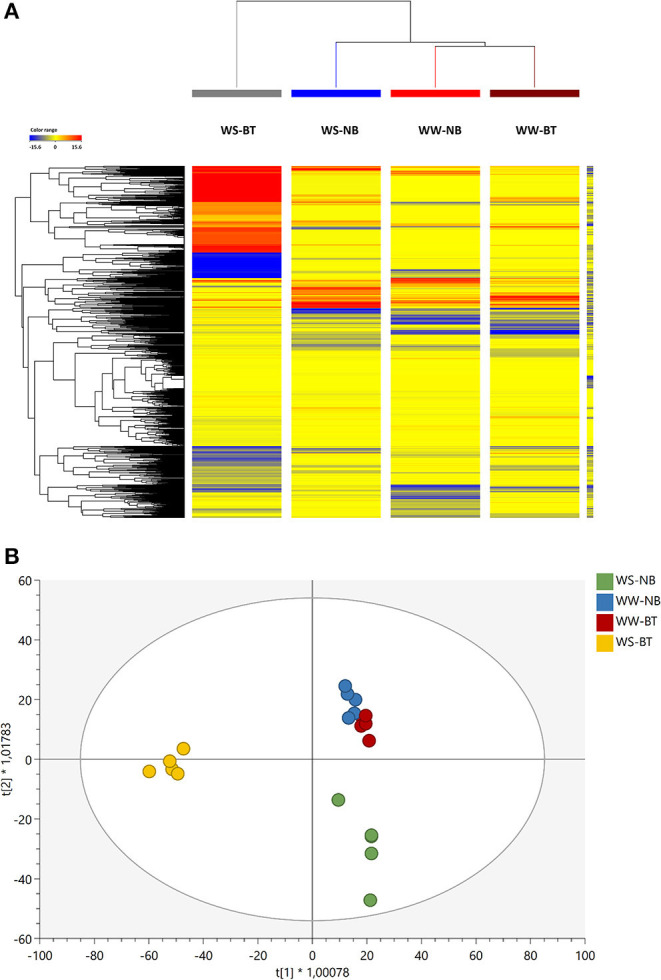
**(A)** Unsupervised hierarchical cluster analysis (HCA, Euclidean similarity; linkage rule: Ward's). The analysis was carried out from metabolite profiles in tomato leaves from the factorial combination of BT and water treatment as gained from ultra-high performance liquid chromatograph (UHPLC) coupled to a quadrupole-time-of-flight mass spectrometer (QTOF-MS) untargeted metabolomics. Compound intensity was used to produce fold-change-based heat maps, on which clustering was based. **(B)** Score plot of Orthogonal Projection to Latent Structures Discriminant Analysis (OPLS-DA) unsupervised analysis carried out from metabolite profiles in tomato leaves treated or untreated plants, as gained from UHPLC/QTOF-MS untargeted metabolomics.

In this study, the untargeted metabolomics approach was able to reveal more than 3,400 molecular features. The annotated compounds and composite mass spectra (mass and abundance combinations), together with compounds confirmed by MS/MS, are listed in Supplementary Table 1. The data set was first interpreted through an unsupervised hierarchical clustering. This unsupervised clustering approach enabled the description of similarities/dissimilarities among treatments, as shown in Figure 5A.

Two main clusters were generated—one including the BT water-stressed treatment and the other including both irrigated and stressed untreated treatments and the treated irrigated one. Two distinct subclusters, one including the untreated water stressed (WS) and the other both the treated and untreated irrigated treatments, could be identified. The WS, BT profile differed starkly from the others: the naive (unsupervised) hierarchical clustering of metabolomic signatures revealed distinctive profiles in tomato leaves under limited water availability, which is the result of the application of the biostimulant.

OPLS-DA analysis allowed separating predictive and orthogonal components (i.e., the components ascribable to technical and biological variation) of variance. The subsequent supervised statistics were used to discriminate the tomato samples according to the treatment. The OPLS-DA (Figure 5B) effectively separated the stressed from the non-stressed plants and pointed out irrigation as the main separation factor. Among the stressed plants, treated plants presented the most distinct profile as suggested by the HCA, and were clearly separated from the non-treated ones. Similar metabolic profiles were found for non-stressed plants regardless of the treatment. The model parameters of the OPLS-DA regression were R2Y = 0.89 and Q2Y = 0.73, respectively. The model was validated (CV-ANOVA *p* = 1.50 × 10^−5^) and overfitting was excluded through permutation testing (*N* = 100). Given the validated model outcomes, the VIP variable selection method was used to identify compounds explaining the observed differences. The discriminating compounds with a VIP score >1.3 were considered as discriminants. About 147 compounds resulted from this selection and are summarized in Supplementary Table 2. Thereafter, a Volcano analysis (*p* < 0.01; FC > 2) was performed and the significant compounds were then uploaded into the Omics Dashboard tool from PlantCyc to facilitate the discussion of results (Supplementary Table 3).

**Figure 6:**
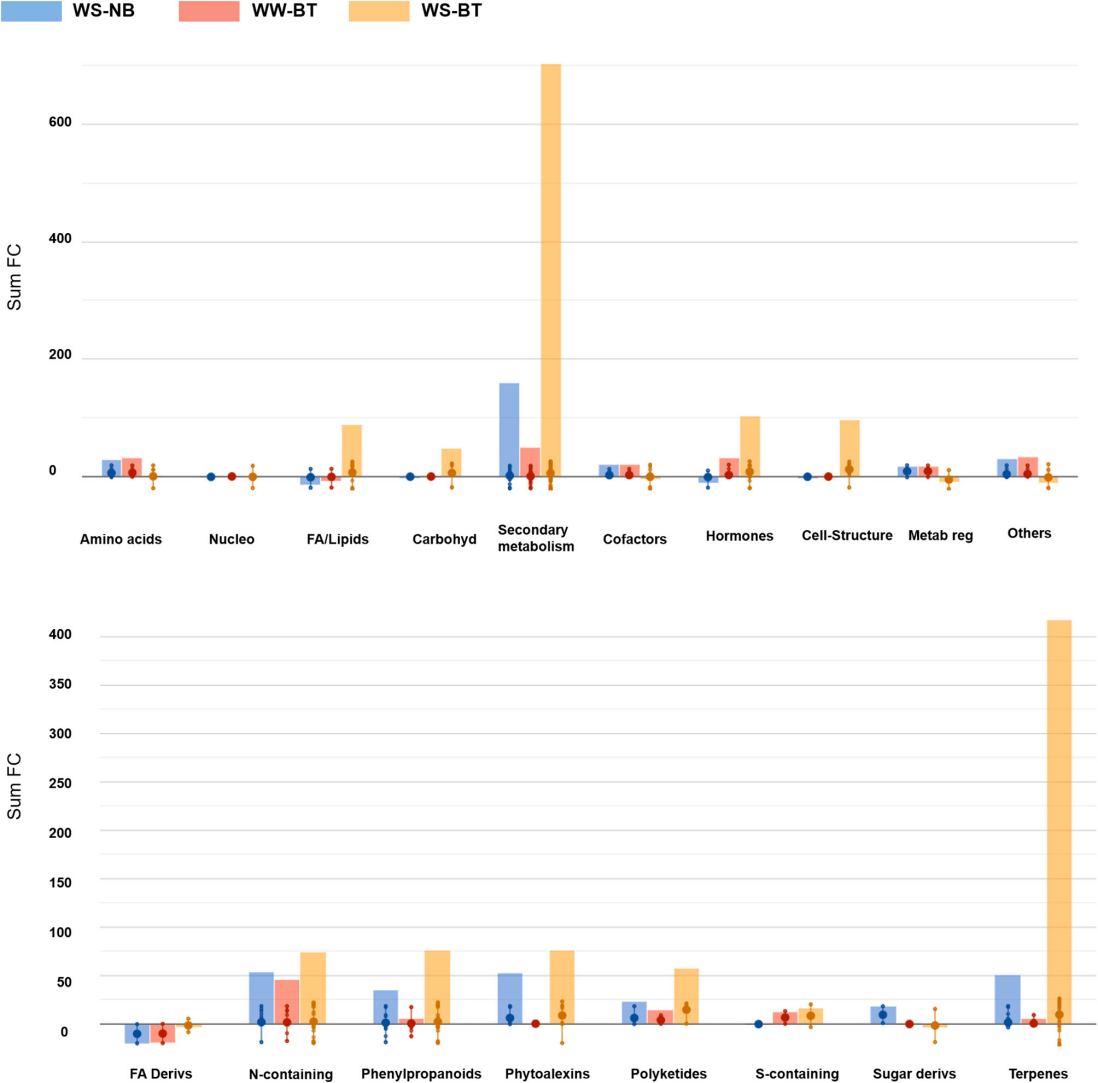
Bar graphs resulting from the import of volcano analysis data [*p* < 0.01; field capacity (FC) > 2] on the Omics Dashboard tool from PlantCyc. The treatments FC difference is calculated based on WW, control treatment (WW–NB). The first bar (in blue) depicts WS–BT results, the second (in red) depicts WW–BT results, the last (in yellow) depicts WS–BT results. The abbreviated subcategory names on the *x*-axis correspond to: Nucleo: nucleosides and nucleotides; FA/Lipids: fatty acids and lipids; Amines: amines and polyamines; Carbohyd: carbohydrates; Cofactors: cofactors, prosthetic groups, electron carriers, and vitamins; Metab reg: Metabolic Regulators.

Notably, relatively few biochemical classes included most of the discriminant metabolites (Figure 6).

The metabolic profile of water-stressed BT plants sharply differs from the others. The most affected classes of compounds were secondary metabolites, particularly in their biosynthesis, followed by cellular structure synthase and fatty acid and lipid synthesis. Regarding secondary metabolite biosynthesis, terpenoid biosynthesis, nitrogen-containing secondary compound biosynthesis, and phenylpropanoid derivative biosynthesis were the most affected. Compounds related to terpenoid biosynthesis were strongly over-accumulated, mostly represented by triterpenoids and tetraterpenoids, with a high abundance of carotenoids: this is particularly relevant considering the role of carotenoids in photosynthetic organisms. At the same time, compounds responsible for terpenoid degradation were significantly underaccumulated. Interestingly, phytoalexins were also found to be over-accumulated. Cell structure synthase metabolites, among which stearate and oleate, were strongly over-accumulated. Concerning vitamin biosynthesis, molecules responsible for thiamine biosynthesis were sharply over-accumulated. Among fatty acids and lipids, cutin synthase metabolites were strongly over-accumulated, together with epoxylated and hydroxylated fatty acids, stearate, and unsaturated fatty acids. Sphingolipids were also found to be over-accumulated. Concerning degradation, amino acids degrading molecules showed a generalized under-accumulation, for instance regarding glutamate, lysine, and tryptophan. Lastly, interestingly gibberellin degrading pathways were under-accumulated in BT plants, but differences could be detected among water treatments: the control group showed a sharp under-accumulation of molecules involved in epoxidation, while the stressed group showed a sharp decrease in succinate content, involved in the hydroxylation of gibberellins.

## Discussion

One of the primary adverse effects of water deficit stress is the inhibition of photosynthesis triggered by stomatal closure, which represents the earliest response to drought (Michaletti et al., 2018). As a result, CO_2_ uptake and concentration in leaves are reduced (Medrano et al., 2002). In view of this, the results from the GAMM analysis confirmed that BT plants performed better in terms of *Pn*, E, and WUE compared to untreated plants under water stress. The use of generalized additive modeling enabled the analysis of the full set of dynamic data, without the need to reduce time resolution (i.e., average overtime or select specific time points). Moreover, with this analysis, the effect of treatments (i.e., drought and biostimulant application) on the patterns (i.e., wiggliness) of gas exchange data was accurately modeled and discriminated, also considering data autocorrelation. The use of GAMM allowed for the extraction of information on the effect of the biostimulant devoid of temporal correlation and random errors due to individual replicates, non-linear interactions between both treatments and the duration of the experiment (expressed in days) and the time of the day (expressed in hours). At the same time, GAMM analysis allowed to consider different smooths for a day (duration of the experiment) and hour (time of the day) for both factors (water and biostimulant). Lastly, the use of GAMM allowed for the inclusion of non-linear variability of the individuals over the duration of the experiment. While the significance of the smooth terms does not provide information on the statistical significance between patterns, each individual variable has a statistically significant influence on modeling the wiggliness of *Pn*, which in turn confirms the distance of *Pn* from a linear function. This resulted in a model explaining 87.8% of the deviance. Further, the use of GAMMs enabled the comparison between the curves of stressed and well-watered plants, both with and without biostimulant, thereby providing both a visual screening tool and a statistical tool to further confirm or disprove the effect of biostimulant treatment. The positive effect of the biostimulant treatment observed through GAMM analysis is in line with literature findings on the potential of GB to increase photosynthetic performance under water stress (Yang and Lu, 2005; Hamani et al., 2020; Nawaz and Wang, 2020), especially in tomato (Mäkelä et al., 1999). Differences in the length of significance windows among *Pn*, E, and WUE were detected. While the photosynthetic rate was constantly higher for treated plants compared to the untreated ones, from stress imposition (39 DAE) to the end of stress (42 DAE), the positive effect in the BT water-stressed plants for transpiration rate, and consequently WUE, was shorter compared to *Pn*. Specifically, under water-stressed conditions, the positive effect of the biostimulant treatment on E was reduced in duration, indicating that higher transpiration could only be supported until late, on 40 DAE (second day of water stress) and efficacy on WUE was further reduced to early on 40 DAE. This indicates that the increased photosynthetic rate after the first day of water stress imposition is followed by increased transpiration, resulting in the reduction of the WUE advantage. WUE is a largely diffused performance indicator for crop yield and water consumption. Water-stressed plants typically exhibit a higher WUE due to a more conservative use of water, resulting in improved resource utilization efficiency under conditions of water scarcity (Zhao et al., 2020). Nevertheless, high stomatal conductance over time is essential to high plant production, translating into maximized soil water-use for transpiration, or effective use of water (EUW) for transpiration. It is therefore evident that, under drought conditions, higher stomatal conductance over time will result in lower WUE (Blum, 2009). Indeed, drought-resistant plants display the minimization of leaf permeability, for example, by means of higher epicuticular wax deposition, in order to maximize soil water capture while diverting water to stomatal transpiration (Blum, 2009). Besides, Blum indicates the EUW as the key objective in maximizing biomass production under a limited water regime.

The increased photosynthetic rate was in line with the protective role of GB in terms of cellular osmotic adjustment, detoxification of reactive oxygen species, and protection and stabilization of membrane integrity (Ashraf and Foolad, 2007) and is further supported by the sharp separation of the metabolic profile of the water-stressed BT thesis. At the same time, the smaller reduction in net photosynthesis in leaves subjected to water stress can be attributed to an increased stomatal conductance, as well as the maintenance of chloroplast ultrastructure (Ma et al., 2006) and Rubisco activity (Yang et al., 2005). Indeed, the overaccumulation of carotenoids is of particular interest, given their photoprotective role in preventing the overexcitation of photosystem II (Uarrota et al., 2018), while at the same time acting as toxic oxygen species scavengers, structure stabilizators, and excess energy dissipators (Griffiths et al., 1955; Frank and Cogdell, 1996; Polívka and Sundström, 2004). GB has indeed been found to have a strong protective effect on the structure and function of the oxygen-evolving complex of PSII *in vitro* against multiple abiotic stresses (Mamedov et al., 1991, 1993; Papageorgiou et al., 1991; Papageorgiou and Murata, 1995; Allakhverdiev et al., 2003). Wang et al. (2010) found that drought stress can interfere with the state of the lipids in thylakoid membrane, which, if damaged, might cause PSII to be impaired. Concurrently, cell membrane stability depends on the absence of lipid peroxidation, caused by reactive oxygen species (ROS) accumulation. Moreover, they found that carotenoid concentrations in water stressed, GB-treated plants were consistently higher compared to untreated plants. They also demonstrated that the ability of GB to decrease ROS levels is not direct, but rather indirect. These findings imply that GB acts as an elicitor to other scavenger molecules, thereby strongly supporting our findings on carotenoid concentration. Indeed, Wang et al. (2010) correlated GB accumulation to xanthophyll cycle-dependent nonradiative energy dissipation, thereby drawing a strong connection between the protective action derived from GB overaccumulation and carotenoid synthesis. In addition, GB-synthetizing transgenic plants have been found to display higher activity of ROS-detoxifying enzymes (Yang et al., 2006; Ahmad et al., 2010). Supporting these arguments, Xu et al. (2018) hypothesized that GB may act both as an osmotic stress hardening molecule and as a signaling molecule in acclimation, rather than only *via* a direct action. Concerning energy supply, it is particularly interesting to notice the sharp accumulation of thiamine precursors, as thiamine plays a pivotal role in carbon metabolism and is essential for cell energy supply in all organisms. Moreover, it is essential in carbon fixation through the Calvin cycle and the non-mevalonate isoprenoid biosynthesis pathway, from which thousands of metabolites are derived including chlorophyll, phytols, and carotenoids, and also several phytohormones (Noordally et al., 2020). Thiamine has also been linked to plant adaptation responses to persistent abiotic stress conditions, including drought (Wong et al., 2006), and oxidative stress (Rapala-Kozik et al., 2008; Tunc-Ozdemir et al., 2009). In addition, the overaccumulation of specific secondary metabolites suggests a stress priming activity induced by the biostimulant treatment: phytoalexins have been associated with increased drought tolerance in multiple species (Kuc, 1995; Hatmi et al., 2014; Vaughan et al., 2015); phenylpropanoid biosynthetic pathway is activated under abiotic stress conditions, resulting in stimulated biosynthesis of phenolic compounds with strong antioxidative potential (Sharma et al., 2019). Lastly, the overaccumulation of lipids and specifically cutin synthase metabolites supports the maximization of stomatal transpiration (Kerstiens, 1997, 2006). All things considered, the positive effect of GB on water stress resistance could be attributed both to the delayed onset of stress, and consequently the enhanced natural response of tomato plants, and the elicitation of stress priming through the induction of H_2_O_2_-mediated antioxidant mechanisms, as Park et al. (2004, 2006) suggested, and molecules with strong antioxidant potential (such as xanthophylls).

In conclusion, the study demonstrated the potential of the GAMM method to describe and discriminate biostimulant action (GB, in this case) to improve photosynthetic performance under water stress conditions. In this, the GAMM method was crucial in extracting the effect of biostimulant treatment under dynamic gas exchange acquisition. GAMM analysis effectively improved the interpretation of time series data, enabling both the description of the dynamics of water stress onset and the isolation of the effect related to the biostimulant treatment. Moreover, compared to the other treatments, water-stressed BT plants displayed a starkly different and stress tolerance-related metabolic profile, in agreement with the findings on photosynthetic performance. The metabolites accumulated suggest a priming effect for stress tolerance, *via* detoxification and stabilization of the photosynthetic machinery. The duration and dynamics of the positive effect of the biostimulant treatment under water stress differed for photosynthesis, transpiration, and WUE, the last two being limited in time. This could depend on an increased transpiration efficiency, translating into maximized soil water use for transpiration, or the EUW for transpiration. At the same time, minimization of leaf permeability through increased leaf wax content is one of the main strategies that plants employ to divert water to stomatal transpiration. Indeed, the metabolic profile findings support the increased EUW through the overaccumulation of lipids and cutine synthase metabolites. The present research brought further evidence that GB protective action on photosystem II is not only direct but also strongly connected to the production of other scavenger molecules (e.g., carotenoids and phytoalexins), making the case that GB acts both as an osmotic stress hardening molecule and as a signaling molecule in acclimation. Nonetheless, further research is needed to deepen the connection between exogenous GB treatment and metabolic response.

## Data Availability Statement

The original contributions presented in the study are included in the article/Supplementary Material, further inquiries can be directed to the corresponding author/s.

## Author Contributions

GA designed and performed the experiment, analyzed, and interpreted the data, created the figures for gas exchange, and wrote the manuscript. GA and MC optimized experimental techniques and analyzed gas exchange data. BM-M performed statistical analysis of the metabolome and data interpretation, created the figures for metabolomics, and participated in methods writing. AF was involved in experimental design and participated in data acquisition. SA supervised the design of the experiment, provided intellectual contributions, oversaw data analysis and interpretation, and corrected the manuscript.

## Conflict of Interest

The authors declare that the research was conducted in the absence of any commercial or financial relationships that could be construed as a potential conflict of interest.

## Supplementary Material

Supplementary Table 1 Untargeted metabolomics data set containing the annotated compounds in both ^***^MS-only and MS/MS following the ultra-high performance liquid chromatography coupled to a quadrupole-time-of-flight mass spectrometer (UHPLC-QTOF-MS) analysis.

Supplementary Table 2 Discriminant metabolites identified by the variables importance in projection (VIP) analysis in leaves following biostimulant application. Compounds were selected as discriminant by possessing a VIP score >1.3.

Supplementary Table 3 Differential metabolites as derived from Volcano analysis [value of *p* < 0.01; field capacity (FC) > 2] and uploaded to the PlantCyc pathway Tool software for the subsequent analysis.
